# Supplementary material for: Complex‐centric proteome profiling by SEC‐SWATH‐MS
Source: Mol Syst Biol. 2019 Jan 14;15(1):e8438. doi: 10.15252/msb.20188438 (PMC6346213; doi:10.15252/msb.20188438)
Supplement: Supplementary file 7 — Dataset EV6 [file MSB-15-e8438-s007.zip › feature_plots_bioplex/Q16186.pdf]

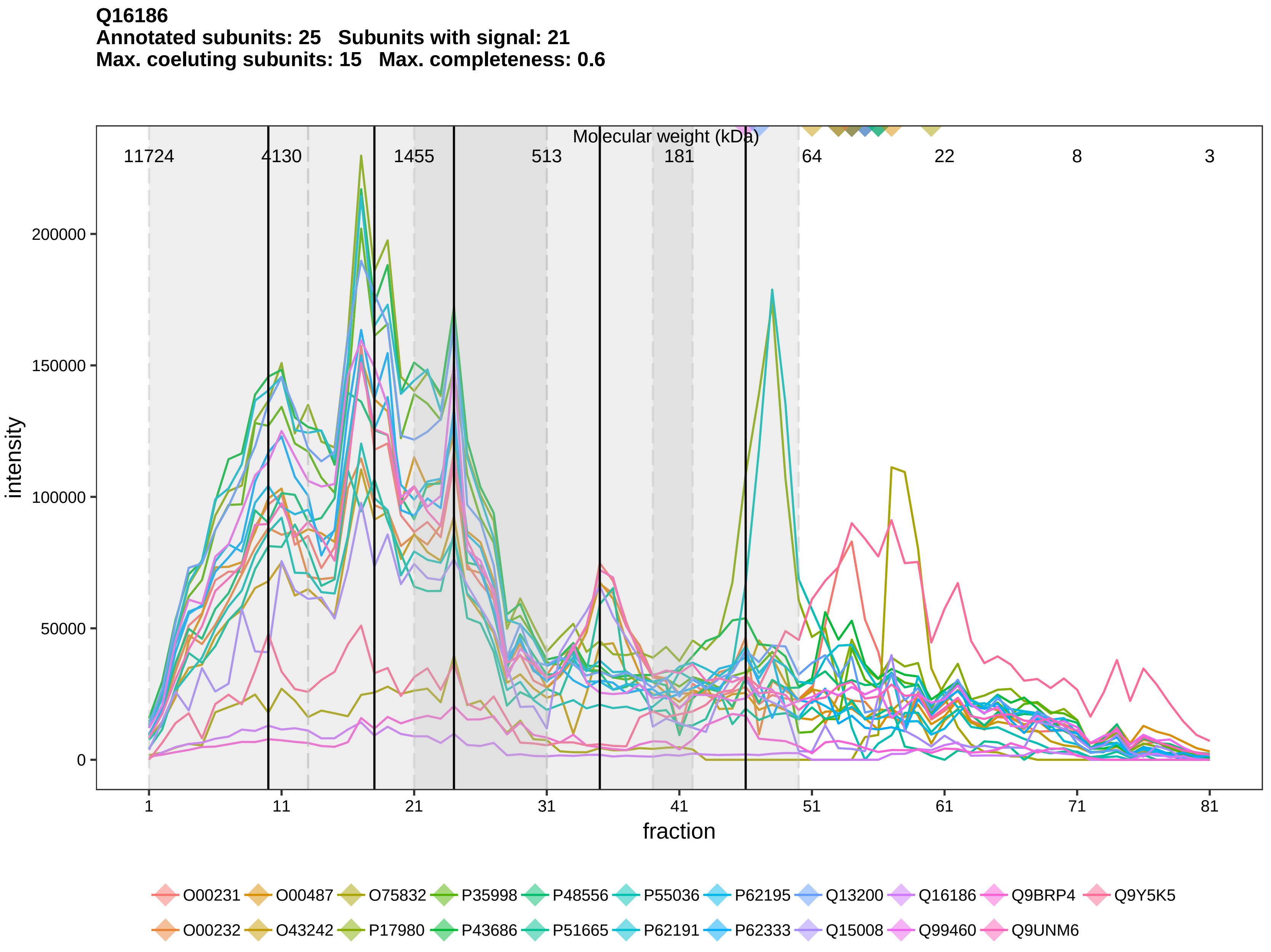

Q16186
Annotated subunits: 25 Subunits with signal: 21
Max. coeluting subunits: 15 Max. completeness: 0.6
Molecular weight (kDa)
11724
4130
1455
513
181
64
22
8
3
intensity
200000
150000
100000
50000
0
fraction
1
11
21
31
41
51
61
71
81
O00231
O00487
O75832
P35998
P48556
P55036
P62195
Q13200
Q16186
Q9BRP4
Q9Y5K5
O00232
O43242
P17980
P43686
P51665
P62191
P62333
Q15008
Q99460
Q9UNM6
